# Inter-individual differences in cognitive function modulate preparatory kinematics during cognitively challenging change-of-direction tasks

**DOI:** 10.3389/fspor.2026.1887464

**Published:** 2026-07-24

**Authors:** Clara Ebner, Matthias Götz, Andrea Kiesel, Dominic Gehring

**Affiliations:** 1 Department of Sport and Sport Science, Exercise and Human Movement Science, University of Freiburg, Freiburg, Germany; 2 Department of Psychology, University of Freiburg, Freiburg, Germany

**Keywords:** ACL, cognitive demands, cognitive function, female football players, preparatory kinematics

## Abstract

**Background:**

Team sport athletes frequently perform changes-of-direction (CODs) in cognitively demanding environments, where necessary preparatory movements may be constrained, potentially increasing the risk for injuries. Inter-individual differences in cognitive function may further modulate these motor-cognitive interactions. Thus, this study examined the influence of inter-individual differences in cognitive function on preparatory kinematics during cognitively demanding COD tasks.

**Methods:**

Twenty-nine female football players completed a computerized cognitive test battery and 90° COD tasks in four conditions with increasing cognitive demand induced by the number of movement options. Preparatory kinematic parameters of lower extremities and trunk were assessed using three-dimensional motion analyses, and linear mixed-model analyses were conducted to examine the effects of cognitive function and cognitive demands.

**Results:**

At the penultimate step, increasing cognitive demands were associated with significantly greater lateral trunk flexion and pelvis tilt, as well as reduced trunk rotation and step width. In addition, interaction effects indicated that better verbal memory scores attenuated the condition effects for lateral trunk flexion [β = −0.52, t(82) = −2.84, *p* = 0.006] and pelvis tilt [β = −0.44, t(82) = −2.55, *p* = 0.01]. At the ultimate step, hip internal rotation and step width significantly increased, while pelvis rotation decreased with increasing cognitive demands. Additionally, longer reaction time was associated with increased lateral trunk flexion towards the cutting leg [β = 2.18, t(24) = 3.08, *p* = 0.005] and increased hip internal rotation [β = 3.90, t(24) = 2.31, *p* = 0.03].

**Conclusion:**

Inter-individual differences in cognitive function, particularly reaction time, modulate preparatory kinematics during cognitively demanding COD tasks in female football players. These findings indicate that cognitive function may influence how athletes adapt preparatory movement strategies under cognitively challenging conditions and highlight the importance of integrating cognitive assessments and preparatory movement analysis when evaluating injury risk and designing targeted prevention programs.

## 1 Background

Team sport athletes are exposed to a rapidly changing and partly unpredictable environment, which is associated with an inherent risk of injury. In football (soccer), changes-of-direction (CODs) are associated with a high risk of non-contact anterior cruciate ligament (ACL) injuries, particularly when being performed in response to external stimuli like an opponent (1). These unanticipated COD tasks therefore lead to substantial demands on movement control and joint stabilization due to uncertainty and limited preparation time. However, ACL injuries are considered multifactorial events. In addition to biomechanical and cognitive factors, anatomical characteristics, neuromuscular control, and other intrinsic and extrinsic factors contribute to ACL injury risk (2). Of note, female athletes exhibit a two- to four-fold higher risk of sustaining an ACL injury compared to their male counterparts, making this population particularly relevant for injury prevention research (3).

To move safely and achieve optimal sport performance in such cognitively challenging environments, athletes rely on cognitive functions that allow the rapid perception, processing and integration of situation- and task-specific information (4, 5). These cognitive functions are assumed to differ inter-individually and include for example reaction time, processing speed, and executive functions (6, 7). Among executive functions, working memory enables the storage and updating of information and, therefore, plays a crucial role in decision-making and the strategic planning of actions (8).

Beyond their importance for sport performance, inter-individual differences in cognitive function of athletes are associated with the risk of sustaining lower limb injuries, particularly regarding the ACL. A recent systematic review indicated that worse baseline cognitive function, specifically reaction time and working memory, was associated with increased musculoskeletal injury risk in team sports (9). In line with this, a case-control study showed that athletes who later sustained an ACL injury had performed significantly worse in a computerized neurocognitive test battery during preseason testing (10). Furthermore, biomechanical studies have shown that longer reaction time, reduced processing speed, and impaired visual memory were related to less favorable, injury-related biomechanical patterns during cognitively demanding movement tasks (11, 12).

Video analyses of ACL injury scenarios during football games revealed that the complexity of cognitive demands shortly before injury occurrence was often high, i.e., the variety of movement options, deceiving actions of an opponent, and limited time to process (visual) information (13). Such scenarios with multiple movement options may place athletes under substantial time pressure, potentially impairing the implementation of appropriate preparatory movements (14). These conditions challenge effective feed-forward control strategies, which are crucial since ACL injuries typically occur rapidly after initial ground contact, well before reactive muscle reflexes can substantially contribute to joint stabilization (15, 16).

Against this background, successful and safe COD execution requires a rapid preparatory reorientation of the body at least one step in advance, i.e., in the penultimate step (17, 18). Substantial evidence suggests that the use of these anticipatory postural adjustment (APA) strategies depends on whether a COD is performed anticipated or unanticipated (17, 19, 20). Specifically, anticipated CODs are typically prepared through a medial foot placement at the penultimate step and controlled trunk motion, whereas unanticipated CODs restrict these strategies and lead to wider steps and excessive trunk and pelvis motion (17, 19). These findings highlight a potential link to the ACL injury mechanism, as changes in trunk kinematics and wide foot placement in preparation of the direction change were accompanied by elevated peak knee abduction moments, a known risk factor for ACL injury (2, 19). Given the time-critical nature of these anticipatory adjustments, it appears plausible that preparatory movement strategies may depend not only on situational constraints but also on inter-individual differences in cognitive function.

Despite a growing number of studies addressing motor–cognitive interactions in the context of ACL injuries (4, 21), evidence remains limited regarding how inter-individual differences in cognitive function influence biomechanics during movement tasks with high cognitive demand. In this context, a recent systematic review highlights the need to compare cognitively demanding tasks with an anticipated movement condition (6). Furthermore, cognitive-motor interference should be analyzed on a continuum rather than categorizing participants into high- and low-performance groups based on baseline cognitive function (4). Finally, previous studies have primarily focused on biomechanical parameters during the stance phase, rather than considering preparatory movements during the penultimate step (4). Consequently, the role of cognitive function in modulating preparatory movement strategies under increasing cognitive demands remains poorly understood.

Therefore, the aim of the study was to examine how inter-individual differences in cognitive function influence trunk, hip and pelvis kinematics during the preparatory phase of a COD task under progressively increasing cognitive demands in female football players. While previous findings from this experimental project demonstrated condition-related biomechanical changes during the ultimate step (22), the current analysis examined how inter-individual differences in cognitive function can serve as a moderating factor specifically addressing preparatory movement strategies including the penultimate step. As the original study was designed and powered to detect condition effects rather than interaction effects, the present moderation analyses should be considered exploratory.

Based on previous literature (4, 14, 19, 20), we hypothesized that increasing cognitive demands would be associated with less favorable preparatory kinematic parameters of proximal segments during penultimate and ultimate steps of COD tasks in female football players. We further hypothesized that better cognitive function would attenuate the effect of increasing cognitive demands on preparatory kinematics and would be associated with more favorable preparatory kinematic patterns across cognitive demands.

## 2 Methods

The present study investigated inter-individual differences in cognitive function and their association with preparatory movement kinematics during a COD task. Participants completed a COD protocol consisting of four conditions with progressively increasing movement-option complexity. Selected biomechanical findings from the experimental project have previously been reported elsewhere (22). The current analyses specifically examine cognitive function as a continuous explanatory variable and focus on preparatory kinematics during the penultimate step of the COD task. The study protocol was preregistered on the Open Science Framework (OSF) and is available at [https://doi.org/10.17605/OSF.IO/4Z5R8]. Further details on the study sample, the experimental setup and data collection are reported in Ebner et al. (2025b) (22) and are here summarized for completeness.

### 2.1 Participants

*A priori* power analyses addressing the aim of detecting condition effects in biomechanical outcomes, as reported in our previous article (22), were based on effect sizes from a similar study (23). The analysis revealed a minimum required sample size of 21 participants to achieve 80% statistical power at an alpha level of 0.05. No separate power analysis was performed for the present secondary analyses of condition×cognitive function interactions. Accordingly, these moderation analyses were considered exploratory.

The final study sample comprised 29 female participants, including 15 high-expertise players (22.9 ± 3.8 years, body height = 1.69 ± 0.05 m, mass = 63.9 ± 5.1 kg, 14.7 ± 3.8 years of club-level playing experience, 8.1 ± 5.1 h of play per week) and 14 low-expertise players (22.5 ± 1.8 years, body height = 1.69 ± 0.06 m, mass = 63.6 ± 5.6 kg, no club-level playing experience). Six of the latter group were engaged in other team sports (e.g., handball and rugby).

All participants were free of lower-limb injuries within the three months preceding study participation. The study was conducted in accordance with the latest version of the Declaration of Helsinki, written informed consent was obtained from all participants, and the protocol was approved by the local ethics committee of the University of Freiburg, Germany (approval number 24-1142-S2, date 24 May 2024).

### 2.2 Cognitive assessment

The cognitive assessment was always conducted before the biomechanical testing, with a break of approximately 30 min between assessments, to avoid potential influences of physical fatigue. Participants completed the *Immediate Post-Concussion Assessment and Cognitive Testing* (ImPACT, version 4.0) unsupervised in a quiet room using a computer and a mouse, which required approximately 20 min (24). The ImPACT is used in sports context to assess athletes' cognitive function (10, 25). In addition to its established use following concussion, it has already been applied to examine motor-cognitive relationships in the context of ACL injuries (10, 12, 26) and other injuries (27). Furthermore, several studies have demonstrated moderate temporal stability of the baseline testing over extended periods in athletes without concussion (28, 29) and have provided evidence for associations with established neurocognitive test batteries (30, 31). Specifically, reported intraclass correlation coefficients (ICCs) indicate moderate to good test–retest reliability of ImPACT composite scores across different retest intervals, with values ranging from 0.60–0.72 for visual memory, 0.65–0.76 for verbal memory, 0.67–0.81 for reaction time, and 0.85–0.88 for visual motor speed (28). Based on the published standard deviations and ICCs reported by Nakayama et al. (2014), the corresponding standard errors of measurement represented approximately 36%–57% of the standard deviations, indicating that measurement error was consistently smaller than the observed between-individual variability in that study (28).

The computer-based test battery consists of six individual subtests, which are aggregated into the four cognitive dimensions visual and verbal memory, visual motor speed and reaction time. Both visual and verbal working memory composite scores were calculated as the average percentage of correct responses across their respective subtests, yielding scores from 0%–100%, with higher values indicating better memory performance (24). Similarly, the visual motor speed score represents a weighted average of the number of correctly executed responses within a fixed time window across two subtests, with higher scores indicating better visual motor speed. The reaction time composite score was calculated as the mean response time for correct answers across three subtests and is reported in seconds, with lower values indicating better reaction ability (24).

### 2.3 Experimental setup and conditions

**Figure 1:**
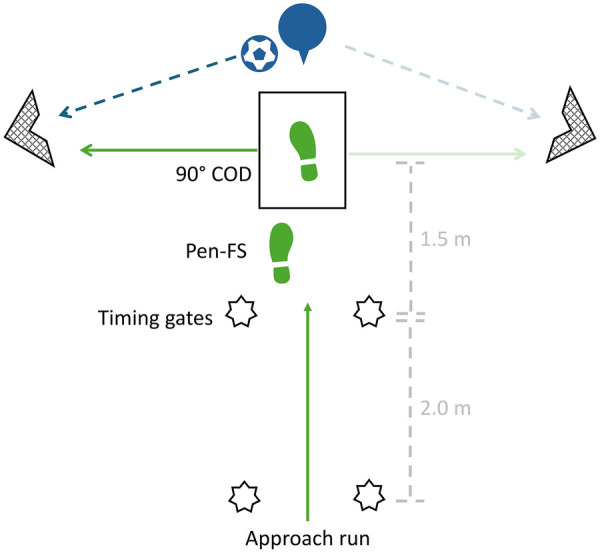
Illustration of the experimental setup using the example of the second condition (UNANT-2) with two movement options, i.e., COD to the left or right. The blue elements represent the opponent and the ball's trajectory, the green parts represent the participant's running direction. Pen-FS, penultimate step foot strike. Ult-FS, ultimate step foot strike on the force plate.

The biomechanical test setup simulates a defensive football-specific scenario, in which the participant adopted the role of a defender, facing a real opponent in ball possession. In each condition, participants performed a submaximal approach run at 4.0 ± 0.3 m/s, followed by a 90° COD to the left or the right in response to a ball that was kicked by the stationary opponent (Figure 1).

During a standardized warm-up and familiarization protocol, participants performed COD trials to both sides and subsequently selected their preferred cutting direction. In all cases, the self-selected preferred direction corresponded to the direction that the investigator qualitatively observed to be more performed with greater movement consistency across familiarization trials. Only trials to this direction were considered for further analyses, whereas the other movement options only served to increase cognitive demands.

Participants completed the movement task in four conditions with increasing levels of cognitive demand, induced by increasing the number of movement options (22). The conditions were performed in a block-randomized order across participants. In the anticipated condition (ANT-1) the COD direction (right or left) was predefined and indicated prior to the approach run. In the unanticipated condition with two movement options (UNANT-2), the participant performed a COD to the left or right in response to the opponent kicking a ball to either side. In the unanticipated condition with three movement options (UNANT-3), the participant performed a COD to either the left of right side or stopped quickly if the opponent did not kick the ball. The unanticipated condition with four movement options (UNANT-4) contained all previous movement options and additionally a deceptive feint by the opponent.

The timing of the stimulus (i.e., ball kick or stop) was standardized, resulting in an available time to react (ATR) of approximately 375 ms, which is considered appropriate to induce an unanticipated condition at comparable approach speeds (32). Each participant performed at least 14 trials per condition, from which five valid trials to the preferred direction were included in further analyses.

### 2.4 Data collection and analysis

A marker-based motion analysis system with 12 cameras (Vicon Motion Systems Ltd., Oxford, Great Britain) was used to collect three-dimensional motion data at 200 Hz. Thirty-seven markers were placed on the participant's head, trunk, legs and shoes. Additional three markers were attached to the ball and 18 markers to the opponent to track their movements. All markers were placed by the same experienced researcher to ensure consistency across participants and minimize inter-rater variability. Following standard procedures for inverse kinematics, joint centers and segments were defined, and kinematics like joint angles were derived using a custom-written script in BodyBuilder (Vicon Motion Systems Ltd., Oxford, UK) as described in detail in Ebner et al. (2025b) (22)).

To extract biomechanical parameters reflecting the preparatory phase of COD execution, two distinct events were defined. First, the foot strike of the ultimate step (ult-FS) was defined as the first frame in which vertical ground reaction force exceeded a value of 20 N, measured using a ground-embedded force plate (AMTI BP600900, Watertown, MA USA), sampling at 1,000 Hz. Second, the foot strike of the penultimate step (pen-FS) was set to the frame, in which the velocity of the toe or heel marker, depending on the foot-strike pattern, dropped below 2.5 m/s along the running direction. This criterion corresponded to the ground reaction force-based identification of ult-FS and was verified in additional analyses to reliably represent ground contact. Specifically, across 119 trials spanning all participants and conditions, the marker velocity-based approach differed from force plate-based foot strike identification by 1.5 ± 6.8 ms on average [range: −15–15 ms], indicating good agreement between both approaches. The Vicon motion capture system and the force plate were hardware-synchronized using a Vicon D-Link system, ensuring temporal alignment of kinematic and kinetic data during acquisition. Subsequent data processing was performed using custom scripts in Matlab (R2022b, The MathWorks Inc.).

Biomechanical outcome parameters were selected based on their relevance to the ACL injury mechanism (1, 2, 17). Trunk and pelvis kinematics of frontal and transverse plane with respect to the global coordinate system as well as step width were extracted at pen-FS and ult-FS (Figure 1). The step width was defined as the medio-lateral distance with respect to the approach run direction from the calcaneus center to the pelvis center. At ult-FS, we additionally extracted hip frontal and transverse plane kinematics as well as knee flexion and abduction angles. Of note, the effects of cognitive demand conditions on ultimate step pelvis, hip and knee kinematics were already analyzed in Ebner et al. (2025b) using ANOVA analyses (22). Importantly, whereas the previous study focused on condition effects and expertise-related differences during the ultimate step, the present work specifically examines whether individual differences in cognitive function moderate the effects of increasing cognitive demands on biomechanical outcomes. In addition, preparatory biomechanics including the penultimate step kinematics were included, representing an additional aspect that was not previously addressed.

The approach run speed was measured by two timing gates (Witty Gate, Microgate, Mahopac, NY, USA), placed at a distance of 3.5 m and 1.5 m from the center of the force plate (Figure 1). Predefined approach speed (4.0 ± 0.3 m/s) as well as available time to react in response to the opponent's (ATR_Opp_, 718 ± 106 ms) and the ball's (ATR_Ball_, 373 ± 108 ms) movement did not differ significantly between conditions (22).

### 2.5 Statistical analyses

Statistical data analyses were performed in R (version 2023.12.0 + 369). The four ImPACT composite scores were z-standardized to facilitate comparability among subtests with varying scales.

Linear mixed-effects models (LMM) were adopted to examine the effects of cognitive function (four z-standardized ImPACT scores) and cognitive demands (four conditions with increasing cognitive demands) on each biomechanical outcome while accounting for within-participant dependencies. Models were fitted using maximum likelihood (ML) estimation in the lme() package. In the first step, the suitability of a LMM for each biomechanical outcome was examined by testing for significant variance between subjects. To this end, a ‘null' model without random intercepts was compared with a second model including random intercepts for participants, using the Akaike information criterion (AIC) and the Bayes information criterion (BIC), with lower values indicating superior fit (33). In cases where the two information criteria disagreed, the model with the lower AIC was selected, as it is preferred for smaller sample sizes and complex models (34). In the next step, *cognitive function*, *cognitive demands* and their interaction (*cognitive function x cognitive demands*) as well as *expertise level* were included as fixed effects. The main effect of *expertise level* did not significantly improve the fit of the models for any of the biomechanical parameters except pelvis tilt at pen-FS and was therefore excluded from the final models. Consistent with this, previous analyses in the same cohort also revealed no associations between expertise level and any of the examined biomechanical parameters during COD tasks (22). Similarly, as the inclusion of random slopes did not improve model fit for any of the biomechanical parameters except trunk rotation at ult-FS, they were omitted from the final models.

Detailed information on the model comparisons is provided in Supplementary A and B.

LMM assumptions were confirmed by visual inspection of histograms (normality), scatter plots (linearity, homoscedasticity, normality of residuals and random intercepts) and by calculating Variance Inflation Factors (multicollinearity). Statistical significance was set at *p* < 0.05. R-squared measures were calculated, with marginal R^2^ representing the variance explained by fixed effects and conditional R^2^ representing the variance explained by both fixed and random effects (35). To further evaluate the relationships among the cognitive predictors, Pearson correlation coefficients among the four ImPACT composite scores were calculated.

## 3 Results

### 3.1 Biomechanics at penultimate step foot strike

**Figure 2:**
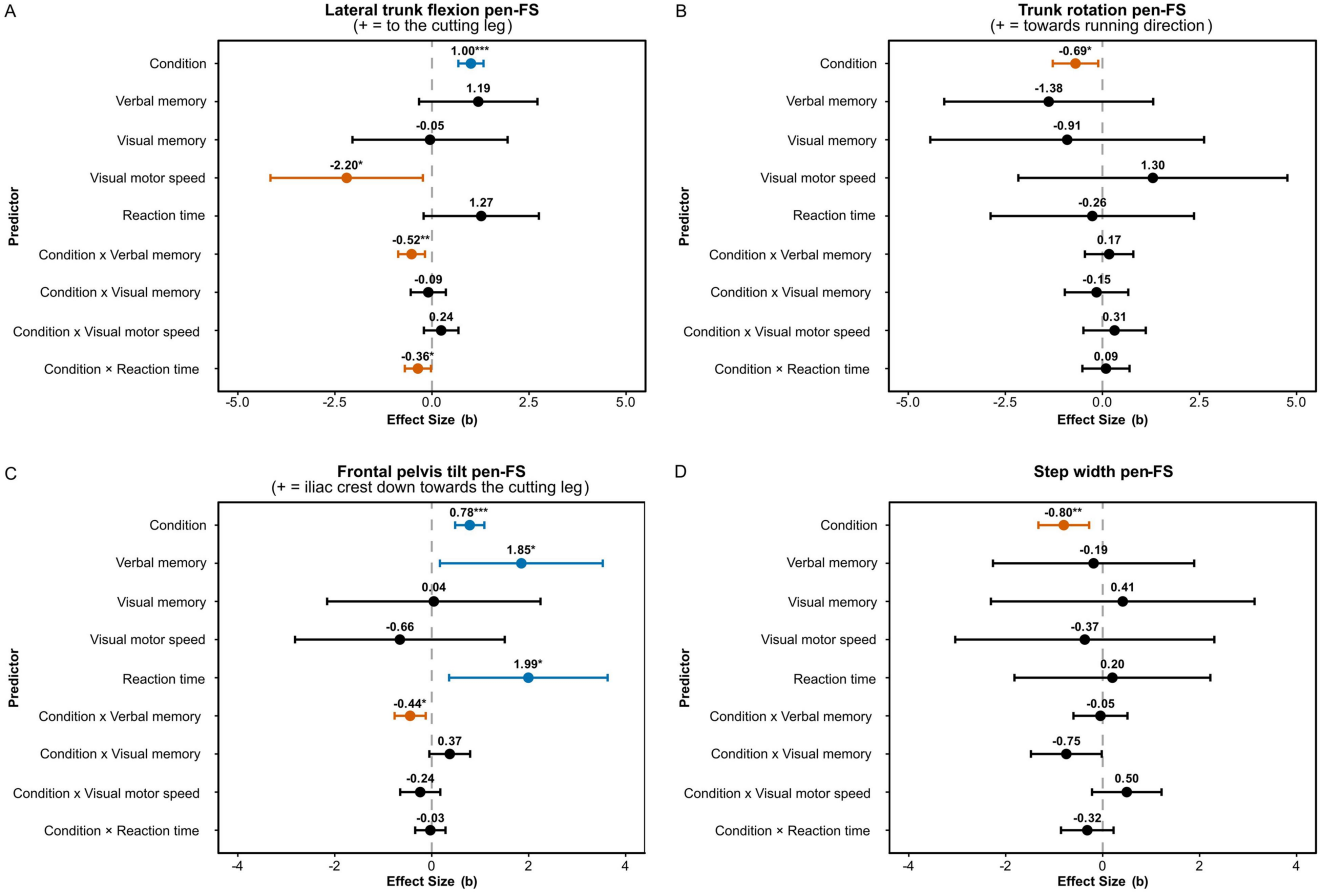
Linear mixed model outputs for selected kinematic parameters at penultimate step foot strike. Red colors indicate significantly negative effects of the respective predictor, blue colors indicate significantly positive effects of the respective predictor, while black indicates no significant effect. The cutting leg refers to the ultimate (plant) foot, i.e., the leg contacting the ground at ultimate step foot strike. **(A)** Lateral trunk flexion. **(B)** Trunk rotation. **(C)** Frontal pelvis tilt. **(D)** Step width. Pen-FS = penultimate step foot strike. * *p* < 0.05, ** *p* < 0.01.

At pen-FS, significant main effects of condition were found for lateral trunk flexion [β = 1.0, t(82) = 5.78, *p* < 0.001; Figure 2A] as well as trunk rotation [β = −0.69, t(82) = −2.13, *p* = 0.04; Figure 2B]. Specifically, with increasing cognitive demands, the trunk was significantly less laterally flexed and rotated toward the new running direction. Additionally, a significant condition effect was found for frontal plane pelvis tilt [β = 0.78, t (82) = 4.79, *p* < 0.001; Figure 2C], indicating reduced tilt towards the new running direction with increasing cognitive demands. Step width at pen-FS decreased with increasing cognitive demands [β = −0.8, t(82) = −2.79, *p* = 0.01; Figure 2D].

Across conditions, significant main effects of cognitive function were identified. Higher verbal memory scores and longer reaction time resulted in increased frontal plane pelvis tilt [β = 1.85, t(24) = 2.46, *p* = 0.02 and β = 1.99, t(24) = 2.73, *p* = 0.01, respectively]. Additionally, higher visual motor speed was associated with reduced lateral trunk flexion at pen-FS across all conditions [β = −2.20, t(24) = −2.44, *p* = 0.02].

Significant condition×verbal memory interactions were found for lateral trunk flexion [β = −0.52, t(82) = −2.84, *p* = 0.006] and frontal plane pelvis tilt [β = −0.44, t(82) = −2.55, *p* = 0.01], indicating that higher verbal memory scores reduced the condition effects. Furthermore, a significant condition×reaction time interaction indicated that longer reaction time significantly attenuated the effect of increased cognitive demands on lateral trunk flexion [β = −0.36, t(82) = −2.01, *p* = 0.047].

No significant main or interaction effects were observed for the remaining kinematic outcomes at pen-FS. Across the models for biomechanical outcomes at pen-FS, marginal R*^2^* values ranged from) 9% (trunk rotation) to 31% (lateral trunk flexion), whereas conditional R*^2^* values ranged from 60% (step width) to 85% (frontal pelvis tilt).

Descriptive statistics of pen-FS kinematics are provided in Table 1. Detailed results of LMM analyses are available in Supplementary D.

**Table 1:** Kinematics at penultimate foot strike (pen-FS): means ± SD.

Kinematic parameters	ANT-1	UNANT-2	UNANT-3	UNANT-4
Lateral trunk flexion [°] (+ = to the cutting leg)	−3.2 ± 4.1	−0.6 ± 3.7	−0.1 ± 4.5	−0.01 ± 3.8
Trunk rotation [°] (+ = towards running direction)	12.1 ± 7.6	10.4 ± 5.7	10.7 ± 6.7	9.7 ± 5.6
Frontal plane pelvis tilt [°] (+ = iliac crest down towards the cutting leg)	−10.0 ± 4.4	−8.7 ± 4.9	−7.7 ± 4.4	−7.8 ± 4.9
Pelvis rotation [°] (+ = towards running direction)	12.2 ± 7.7	12.2 ± 8.8	11.2 ± 8.4	11.3 ± 8.0
Step width [cm]	24.6 ± 4.0	23.3 ± 5.4	22.0 ± 4.6	22.4 ± 5.3

ANT, anticipated, ATR, available time to react, UNANT, unanticipated.

### 3.2 Biomechanics at ultimate foot strike

**Figure 3:**
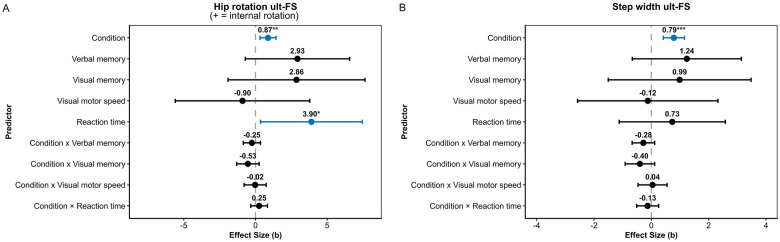
Linear mixed model outputs for selected biomechanical parameters at ultimate step foot strike. Red colors indicate significantly negative effects of the respective predictor, blue colors indicate significantly positive effects of the respective predictor, while black indicates no significant effect. The cutting leg refers to the ultimate (plant) foot, i.e., the leg contacting the ground at ultimate step foot strike. **(A)** Hip rotation. **(B)** Step width. Ult-FS = ultimate step foot strike. *p < 0.05, **p < 0.01.

At ult-FS, significant main effects of the condition were found for lateral trunk flexion [β = 0.76, t(82) = 4.19, *p* < 0.001] and frontal plane pelvis tilt [β = 0.55, t(82) = 3.29, *p* = 0.001], with reduced orientation toward the new running direction with increasing cognitive demands. Additionally, significant condition effects indicated reduced pelvis rotation [β = −1.17, t(82) = −3.02, *p* = 0.003] toward the new running direction and increased hip internal rotation [β = 0.87, t(82) = 2.83, *p* = 0.01; Figure 3A] with increasing cognitive demands. Step width at ult-FS also increased significantly with increasing cognitive demands [β = 0.79, t(82) = 4.05, *p* = <0.001; Figure 3B].

Regarding the main effects of cognitive function, across all conditions longer reaction times were associated with increased lateral trunk flexion [β = 2.18, t(24) = 3.08, *p* = 0.005] and greater pelvis tilt [β = 2.29, t(24) = 2.73, *p* = 0.01]. Additionally, longer reaction times were associated with increased hip internal rotation [β = 3.90, t(24) = 2.31, *p* = 0.03] at ult-FS.

No significant condition x cognitive function interactions were observed for kinematic parameters at ult-FS (*p* > 0.05). Moreover, no main effects were found for knee kinematics at ult-FS (*p* > 0.05).

Please note that the results regarding pelvis, hip kinematics and knee flexion angles at ult-FS were already reported in Ebner et al. (2025b) (22); however, those analyses were based on ANOVA models and did not include cognitive function as an additional predictor.

Across the models for biomechanical outcomes at ult-FS, marginal R^2^ values ranged from) 5% (hip abduction) to 29% (frontal pelvis tilt and hip internal rotation), whereas conditional R^2^ values ranged from 76% (knee flexion) to 93% (knee abduction).

Descriptive statistics for ult-FS kinematics are provided in Table 2. Detailed results of LMM analysis are available in Supplementary E.

**Table 2:** Kinematics at ultimate step IC (ult-FS): means ± SD.

Kinematic parameters	ANT-1	UNANT-2	UNANT-3	UNANT-4
Lateral trunk flexion [°] (+ = to the cutting leg)	−2.5 ± 4.1	−1.2 ± 4.4	−0.4 ± 4.7	−0.2 ± 4.2
Trunk rotation [°] (+ = towards running direction)	3.2 ± 11.4	3.4 ± 8.3	2.3 ± 8.3	2.3 ± 8.0
Frontal plane pelvis tilt [°] (- = iliac crest down towards the cutting leg)	−11.2 ± 5.0	−10.0 ± 5.6	−9.6 ± 4.7	−9.5 ± 5.4
Pelvis rotation [°] (+ = towards running direction)	22.9 ± 10.5	20.5 ± 9.4	20.2 ± 7.9	19.1 ± 9.0
Hip abduction [°] (+ = abduction)	9.0 ± 4.9	9.8 ± 5.0	9.9 ± 4.3	9.7 ± 5.2
Hip internal rotation [°] (+ = internal rotation)	4.8 ± 10.0	6.1 ± 10.7	7.1 ± 10.2	7.4 ± 10.4
Knee flexion [°] (+ = flexion)	27.0 ± 6.8	27.9 ± 7.6	28.3 ± 8.1	27.5 ± 7.0
Knee abduction [°] (+ = abduction)	7.7 ± 3.9	7.9 ± 3.6	7.7 ± 3.8	8.3 ± 3.5
Step width [cm]	26.6 ± 4.8	28.9 ± 4.5	28.4 ± 4.7	29.4 ± 4.1

ANT, anticipated, UNANT, unanticipated.

Intercorrelations among the four ImPACT composite scores are presented in Supplementary C. Correlations ranged from r = −0.15 to r = 0.67, with the only significant association observed between verbal memory and visual motor speed (*p* < 0.001). Variance inflation factors ranged from 1.00 to 2.76, indicating no evidence of problematic multicollinearity among the predictors included in the models (33).

## 4 Discussion

The present work used an experimental cohort previously described in Ebner et al. (22) to examine how inter-individual differences in cognitive function influence preparatory biomechanics during a football-specific COD task under increasing cognitive demands. The main findings are that increasing cognitive demands altered preparatory trunk, pelvis, and hip kinematics, while inter-individual differences in cognitive function, especially reaction time and verbal memory, moderated these biomechanical responses.

### 4.1 Effects of cognitive demands and cognitive function on proximal segments kinematics

In the present study, increasing cognitive demands were associated with greater lateral pelvis tilt and lateral trunk flexion towards the cutting leg, as well as reduced trunk rotation toward the new running direction at both pen-FS and ult-FS. These findings are in line with previous studies demonstrating that increasing cognitive demands lead to increased pelvis tilt and lateral trunk flexion as well as reduced trunk rotation during COD tasks (19, 20, 36).

These effects are also consistent with the second of two primary APA movement strategies that have been reported during the penultimate step to prepare safe and successful COD execution (14, 17, 19): (1) frontal plane foot placement and (2) frontal and transverse plane trunk and pelvis motion, with the latter being dominantly used under unanticipated, time-constraint conditions (2, 17, 19). From a mechanical perspective, previous studies have suggested that frontal plane trunk motion, mediated by pelvis control, may contribute to center of mass acceleration and redirection due to the large mass of the trunk segment (14, 17). Likewise, trunk and pelvis rotation have been associated with an earlier orientation toward the new running direction (19). However, lateral trunk flexion toward the cutting leg has previously been associated with increased ACL loading, potentially through alterations in ground reaction force application relative to the knee joint (37). Furthermore, previous studies identified lateral trunk flexion as a predictor of knee abduction moments, explaining approximately 17%–18% of their variance (19, 37). However, these mechanisms were not directly assessed in the present study and should therefore be regarded as potential interpretations rather than direct consequences of the observed kinematic changes.

The cognitive demands effects for kinematics of proximal segments might be explained by an increase in the choice reaction time due to increasing movement options, which is known as Hick's law (38). The role of restricted reaction time as a primary contributor to cognitive demands is supported by the findings of a previous study, where the systematic reduction of reaction times was associated with increasing lateral trunk flexion and knee abduction moments (19).

Beyond these general condition effects, the present exploratory analyses suggest that inter-individual differences in cognitive function may modulate preparatory control during COD tasks. Specifically, higher verbal memory scores were associated with a tendency toward a smaller increase in lateral trunk flexion and pelvis tilt at pen-FS as cognitive demands increased. However, given the exploratory nature of the present moderation analyses, these findings should be interpreted cautiously and require confirmation in adequately powered future studies. Similarly, Giesche et al. (2020) reported fewer landing errors during reactive jump-landing tasks in athletes with better working memory (39). Although verbal memory and working memory are distinct cognitive constructs, previous validation studies have reported associations between ImPACT memory composites and established neurocognitive tests (30, 31). Therefore, some overlap between the underlying cognitive processes cannot be excluded. As working memory was not directly assessed in the present study, the observed association between verbal memory and preparatory biomechanics should be interpreted as an exploratory finding that requires further verification.

Moreover, in a recent systematic review, Bertozzi et al. (2023) reported that five of six studies associated worse cognitive function with riskier ACL-related biomechanics (4). The present findings are generally consistent with this finding and suggest that such associations may already be reflected in preparatory kinematics preceding ultimate ground contact. However, given the restriction to predefined movement options and preferred-direction trials in the study design, these results should be interpreted in the context of experimentally imposed cognitive demands rather than match-play decision-making in football.

Contrary to our expectations, longer reaction times, i.e., worse performance, attenuated the effects of increasing cognitive demands on lateral trunk flexion at pen-FS. One possible explanation is that athletes with slower reaction times may adopt a more conservative movement strategy under increasing task demands, thereby reducing further modulation of trunk motion. However, this interpretation remains speculative and contrasts with the overall pattern of greater biomechanical alterations with increasing cognitive demands for individuals with longer reaction times. To examine the possibility that this finding was driven by suppression due to the inclusion of the other ImPACT domains, we conducted an additional analysis in which these variables were removed from the model, but the condition x reaction time interaction effect remained of comparable magnitude and direction. Furthermore, this interaction effect was associated with a *p*-value close to the significance threshold (*p* = 0.047), while the corresponding 95% confidence interval was located close to zero [95% CI (−0.70, −0.02)], indicating limited precision of the estimate. Therefore, particularly in light of the number of interaction tests conducted across outcome measures, the possibility of a Type I error cannot be excluded. Consequently, this finding should be interpreted with caution.

In addition to interaction effects, several cognitive function domains demonstrated main effects independent of cognitive demand levels. At pen-FS, a higher visual motor speed score was associated with reduced lateral trunk flexion, and longer reaction time was associated with increased pelvis tilt. At ult-FS, longer reaction time was associated with increased lateral trunk flexion and pelvis tilt across all conditions. A similar, albeit less pronounced, association was observed for hip internal rotation. These findings indicate that longer reaction times may constrain the organization of COD movement strategies by delaying motor planning and limiting the time available for feed-forward control, regardless of cognitive demand conditions (11). Notably, nine out of 29 participants in our study demonstrated “low average” reaction times, whereas nearly all participants performed average or above average on the remaining ImPACT domains (24). This finding may help explain why associations with biomechanical modifications were most pronounced for the reaction time composite score, and less so for the other cognitive domains.

Of note, we found no main or interaction effects for knee flexion or knee abduction angles at ult-FS. These findings contrast with some previous studies that found significant effects of cognitive function on knee mechanics during jump-landing and cutting (11, 12, 40). Differences in movement tasks, ecological validity, or cognitive demand characteristics might account for these divergent findings.

### 4.2 Effects of cognitive demands and cognitive function on foot placement strategies

In the present study, step width at pen-FS decreased with increasing cognitive demands. From a mechanical perspective, frontal plane foot placement determines the ground reaction force application point and thereby the direction and magnitude of the ground reaction force acting on the center of mass (41). A more medial foot placement in the penultimate step facilitates the redirection in the ultimate step, whereas a wider foot placement increases the acceleration of the center of mass against the desired direction (17). However, the observed reduction in step width, i.e., the more medial foot placement, at pen-FS contradicts earlier findings (17, 20, 36). A likely explanation is the short transition from pen-FS to ult-FS typically (0.14 ± 0.05 s) in the present task without a clear flight phase. This markedly reduced temporal separation between steps may have challenged the participants' ability to apply APA strategies relying on foot placement, and might therefore limit comparability with studies reporting a more distinct preparatory step (14).

At ult-FS, step width increased under unanticipated conditions, which is in line with a previous study, suggesting that increased lateral trunk flexion and rotation away from the new running direction in unanticipated CODs requires wider step width to permit redirection (20). Importantly, step width at pen-FS and ult-FS may not be fully independent, as foot placement during the penultimate step could influence the positioning of the subsequent ultimate step (17). However, no main or interaction effects of cognitive function were observed for step width at pen-FS or ult-FS. These findings suggest that cognitive function may have less influence on foot placement strategy than on proximal segment kinematics.

### 4.3 Practical implications

This study offers several potential practical implications. First, it should be considered to conduct biomechanical assessments under cognitively demanding conditions, as deficits in movement control may only become apparent in such situations. Importantly, preparatory movements during the penultimate step and at ultimate step foot strike should be included, as cognitive influences on potentially unfavorable movement patterns were already evident at this early timepoint.

Second, the LMM analyses with high conditional R^2^ for all outcome parameters (Supplementary D and E) indicated substantial interindividual variability. Therefore, future injury-risk screening approaches may benefit from incorporating cognitive assessments alongside biomechanical analyses. Whether computer-based test batteries such as the ImPACT can contribute to injury-risk screening remains to be determined in prospective studies. Finally, future studies should investigate whether interventions targeting cognitive function can positively influence movement control under cognitively demanding conditions. It is important to remark that all these implications should be interpreted cautiously given the relatively small absolute differences between conditions and the observational nature of the present findings.

### 4.4 Limitations

This study is not without limitations. A key strength of this study is the use of an ecologically valid instead of artificial visual stimulus, but this also introduces temporal variability. Although the ATR did not differ significantly between conditions, it depended largely on whether the subjects reacted to a movement of the opponent or the ball (22).

The sample size was originally calculated to detect condition effects on ultimate step biomechanics (22). However, the present analyses additionally examined condition×cognitive function interaction effects. Because interaction effects generally require larger sample sizes, the present sample with 29 participants may have limited the ability to detect small-to-moderate interaction effects. Therefore, estimates of interaction effects should be interpreted with caution and findings should be considered exploratory and warrant replication in larger independent samples.

Another limitation relates to the large number of tested main and interaction effects across multiple biomechanical outcome variables, which increases the risk of Type I errors. Consequently, findings with *p*-values close to the significance threshold (e.g., condition x reaction time interaction for lateral trunk flexion at pen-FS) should be interpreted with caution, as some effects may not remain statistically significant after correction for multiple comparisons and the present moderation analyses should generally be regarded as exploratory. Similarly, although several effects reached statistical significance, the observed absolute differences in segment angles were generally small. As no established thresholds for meaningful changes in these variables currently exist, cautious interpretation of the practical relevance of these findings is necessary.

Moreover, the assessment of cognitive function using a computer-based cognitive test battery might have diminished the transferability of the results to game situations. While the ImPACT was used to capture individual differences in general cognitive function, it does not directly assess football-specific perceptual-cognitive abilities such as anticipation of opponents' actions or decision-making in dynamic environments. Supporting this notion, a previous study reported only low to moderate construct validity of computer-based measures of inhibition and visuospatial working memory measured against sport-specific tests for instance on a SpeedCourt system (42). Therefore, the present findings should not be interpreted as reflecting sport-specific cognitive performance. Future studies might consider more ecologically valid assessment tools for cognitive function to better reflect the perceptual and decision-making demands of football. Furthermore, although published evidence (28) indicates moderate to good temporal stability of the ImPACT composite scores used in the present study, the battery was originally developed for within-subject change detection following concussion. Consequently, state-dependent influences and measurement error should be considered when interpreting between-individual differences in cognitive performance.

Because biomechanical analyses were restricted to the preferred cutting direction, the findings may not directly generalize to the non-preferred cutting direction of the participants. Future studies might therefore examine whether the observed cognitive-biomechanical relationships are consistent across different movement options and should additionally consider the potential influence of limb dominance on movement control under cognitively demanding conditions. Finally, the present study included only female football players. Therefore, the findings cannot be directly generalized to male athletes or other sporting populations. Although previous studies in male athletes have also reported associations between cognitive function and movement biomechanics (12, 39), it remains unclear whether the magnitude and nature of these relationships differ between sexes. Accordingly, potential sex-specific differences warrant further investigation.

## 5 Conclusions

The present study suggests that inter-individual differences in cognitive function, particularly reaction time and verbal memory, may influence the biomechanical effects of increasing cognitive demands during COD tasks in female football players. These associations were already evident during preparatory movements of the penultimate and ultimate step foot strikes. Accordingly, evaluating movement control under cognitively demanding conditions and considering preparatory movement phases may provide additional insight into individual movement strategies associated with ACL injuries. Given that cognitive function was assessed using a computer-based test battery rather than sport-specific tests, further research is required to establish the practical relevance of cognitive testing and its potential implication for injury-risk screening and prevention.

## Data Availability

The raw data supporting the conclusions of this article will be made available by the authors, without undue reservation.
