# Supplementary material for: Inter-individual differences in cognitive function modulate preparatory kinematics during cognitively challenging change-of-direction tasks
Source: Front Sports Act Living. 2026 Jul 24;8:1887464. doi: 10.3389/fspor.2026.1887464 (PMC13447175; doi:10.3389/fspor.2026.1887464)
Supplement: Supplementary file 1 [file Datasheet1.docx]

**Supplement A: Comparison of linear mixed models for kinematics at penultimate step foot strike (pen-FS).**

| **Dependent variable** | **Model** | **Fixed Effects** | **Random Effects** | **Fitting parameters** | | | **Model comparison** | | | |
| --- | --- | --- | --- | --- | --- | --- | --- | --- | --- | --- |
|  |  |  |  | **AIC** | **BIC** | **LL** | **Number of comparison model** | **df** | **LL-ratio** | |
| Lateral trunk flexion [°] | 1 | - | - | 664.8 | 670.3 | -330.4 | - | - | | - |
|  | 2 | - | Intercepts | 609.0 | 617.2 | -301.5 | 1 | 3 | | 57.8** |
|  | 3 | Condition, Verbal memory, Visual memory, Visual motor speed, Reaction time, Condition x Verbal memory, Condition x Visual memory, Condition x Visual motor speed, Condition x Reaction time | Intercepts | 575.0 | 608.1 | -275.5 | 2 | 12 | | 51.9** |
|  | 4 | Condition, Verbal memory, Visual memory, Visual motor speed, Reaction time, Condition x Verbal memory, Condition x Visual memory, Condition x Visual motor speed, Condition x Reaction time, Expertise | Intercepts | 576.8 | 612.6 | -275.4 | 3 | 13 | | 0.2 |
|  | 5 | Condition, Verbal memory, Visual memory, Visual motor speed, Reaction time, Condition x Verbal memory, Condition x Visual memory, Condition x Visual motor speed, Condition x Reaction time | Intercepts & Slopes | 577.9 | 616.5 | -275.0 | 3 | 14 | | 1.1 |
| Trunk rotation [°] | 1 | - | - | 764.2 | 769.7 | -380.1 | - | - | | - |
|  | 2 | - | Intercepts | 701.6 | 709.9 | -347.8 | 1 | 3 | | 64.5** |
|  | 3 | Condition, Verbal memory, Visual memory, Visual motor speed, Reaction time, Condition x Verbal memory, Condition x Visual memory, Condition x Visual motor speed, Condition x Reaction time | Intercepts | 710.1 | 743.2 | -343.1 | 2 | 12 | | 9.5 |
|  | 4 | Condition, Verbal memory, Visual memory, Visual motor speed, Reaction time, Condition x Verbal memory, Condition x Visual memory, Condition x Visual motor speed, Condition x Reaction time, Expertise | Intercepts | 708.6 | 749.9 | -339.3 | 3 | 15 | | 7.6 |
|  | 5 | Condition, Verbal memory, Visual memory, Visual motor speed, Reaction time, Condition x Verbal memory, Condition x Visual memory, Condition x Visual motor speed, Condition x Reaction time | Intercepts & Slopes | 710.6 | 749.1 | -341.3 | 3 | 14 | | 3.6 |
| Frontal pelvis tilt [°] | 1 | - | - | 690.2 | 695.7 | -343.1 | - | - | | - |
|  | 2 | - | Intercepts | 594.6 | 602.9 | -294.3 | 1 | 3 | | 97.5** |
|  | 3 | Condition, Verbal memory, Visual memory, Visual motor speed, Reaction time, Condition x Verbal memory, Condition x Visual memory, Condition x Visual motor speed, Condition x Reaction time | Intercepts | 572.1 | 605.2 | -274.1 | 2 | 12 | | 40.5** |
|  | 4 | Condition, Verbal memory, Visual memory, Visual motor speed, Reaction time, Condition x Verbal memory, Condition x Visual memory, Condition x Visual motor speed, Condition x Reaction time, Expertise | Intercepts | 568.8 | 604.6 | -271.4 | 3 | 13 | | 5.3* |
|  | 5 | Condition, Verbal memory, Visual memory, Visual motor speed, Reaction time, Condition x Verbal memory, Condition x Visual memory, Condition x Visual motor speed, Condition x Reaction time | Intercepts & Slopes | 573.9 | 612.5 | -273.0 | 4 | 14 | | 2.2 |
| Pelvis rotation [°] | 1 | - | - | 819.3 | 824.9 | -407.7 | - | - | | - |
|  | 2 | - | Intercepts | 774.5 | 782.7 | -384.2 | 1 | 3 | | 46.9** |
|  | 3 | Condition, Verbal memory, Visual memory, Visual motor speed, Reaction time, Condition x Verbal memory, Condition x Visual memory, Condition x Visual motor speed, Condition x Reaction time | Intercepts | 780.8 | 813.8 | -378.4 | 2 | 12 | | 11.7 |
|  | 4 | Condition, Verbal memory, Visual memory, Visual motor speed, Reaction time, Condition x Verbal memory, Condition x Visual memory, Condition x Visual motor speed, Condition x Reaction time, Expertise | Intercepts | 782.7 | 818.5 | -378.3 | 3 | 13 | | 3.5 |
|  | 5 | Condition, Verbal memory, Visual memory, Visual motor speed, Reaction time, Condition x Verbal memory, Condition x Visual memory, Condition x Visual motor speed, Condition x Reaction time | Intercepts & Slopes | 781.2 | 819.8 | -376.6 | 3 | 14 | | 3.5 |
| Step width (cm) | 1 | - | - | 701.0 | 706.5 | -348.5 | - | - | | - |
|  | 2 | - | Intercepts | 668.4 | 676.7 | -331.2 | 1 | 3 | | 34.6** |
|  | 3 | Condition, Verbal memory, Visual memory, Visual motor speed, Reaction time, Condition x Verbal memory, Condition x Visual memory, Condition x Visual motor speed, Condition x Reaction time | Intercepts | 669.2 | 702.2 | -322.6 | 2 | 12 | | 17.3* |
|  | 4 | Condition, Verbal memory, Visual memory, Visual motor speed, Reaction time, Condition x Verbal memory, Condition x Visual memory, Condition x Visual motor speed, Condition x Reaction time, Expertise | Intercepts | 669.5 | 705.3 | -321.7 | 3 | 13 | | 1.7 |
|  | 5 | Condition, Verbal memory, Visual memory, Visual motor speed, Reaction time, Condition x Verbal memory, Condition x Visual memory, Condition x Visual motor speed, Condition x Reaction time | Intercepts & Slopes | 670.2 | 708.7 | -321.1 | 3 | 14 | | 3.0 |
| * p < 0.05, ** p < 0.001 | | | | | | | | | | |

**Supplement B: Comparison of linear mixed models for kinematics at ultimate step foot strike (ult-FS).**

| **Dependent variable** | **Model** | **Fixed Effects** | **Random Effects** | **Fitting parameters** | | | **Model comparison** | | | |
| --- | --- | --- | --- | --- | --- | --- | --- | --- | --- | --- |
|  |  |  |  | **AIC** | **BIC** | **LL** | **Number of comparison model** | **df** | **LL-ratio** | |
| Lateral trunk flexion [°] | 1 | - | - | 674.8 | 680.3 | -335.4 | - | - | | - |
|  | 2 | - | Intercepts | 600.2 | 608.2 | -297.1 | 1 | 3 | | 76.6** |
|  | 3 | Condition, Verbal memory, Visual memory, Visual motor speed, Reaction time, Condition x Verbal memory, Condition x Visual memory, Condition x Visual motor speed, Condition x Reaction time | Intercepts | 586.6 | 619.6 | -281.3 | 2 | 12 | | 31.6** |
|  | 4 | Condition, Verbal memory, Visual memory, Visual motor speed, Reaction time, Condition x Verbal memory, Condition x Visual memory, Condition x Visual motor speed, Condition x Reaction time, Expertise | Intercepts | 586.6 | 622.4 | -280.3 | 3 | 13 | | 2.0 |
|  | 5 | Condition, Verbal memory, Visual memory, Visual motor speed, Reaction time, Condition x Verbal memory, Condition x Visual memory, Condition x Visual motor speed, Condition x Reaction time | Intercepts & Slopes | 588.5 | 627.1 | -280.3 | 3 | 14 | | 2.04 |
| Trunk rotation [°] | 1 | - | - | 842.3 | 847.8 | -419.1 | - | - | | - |
|  | 2 | - | Intercepts | 765.3 | 773.6 | -379.6 | 1 | 3 | | 79.0** |
|  | 3 | Condition, Verbal memory, Visual memory, Visual motor speed, Reaction time, Condition x Verbal memory, Condition x Visual memory, Condition x Visual motor speed, Condition x Reaction time | Intercepts | 773.2 | 806.3 | -374.6 | 2 | 12 | | 10.1 |
|  | 4 | Condition, Verbal memory, Visual memory, Visual motor speed, Reaction time, Condition x Verbal memory, Condition x Visual memory, Condition x Visual motor speed, Condition x Reaction time, Expertise | Intercepts | 768.7 | 810.0 | -369.3 | 3 | 15 | | 2.4 |
|  | 5 | Condition, Verbal memory, Visual memory, Visual motor speed, Reaction time, Condition x Verbal memory, Condition x Visual memory, Condition x Visual motor speed, Condition x Reaction time | Intercepts & Slopes | 769.1 | 807.6 | -370.5 | 3 | 14 | | 8.1* |
| Frontal pelvis tilt [°] | 1 | - | - | 713.6 | 719.1 | -354.8 | - | - | | - |
|  | 2 | - | Intercepts | 590.6 | 598.8 | -292.3 | 1 | 3 | | 125.1** |
|  | 3 | Condition, Verbal memory, Visual memory, Visual motor speed, Reaction time, Condition x Verbal memory, Condition x Visual memory, Condition x Visual motor speed, Condition x Reaction time | Intercepts | 584.9 | 617.9 | -280.4 | 2 | 12 | | 23.7* |
|  | 4 | Condition, Verbal memory, Visual memory, Visual motor speed, Reaction time, Condition x Verbal memory, Condition x Visual memory, Condition x Visual motor speed, Condition x Reaction time, Expertise | Intercepts | 584.2 | 520.0 | -279.1 | 3 | 13 | | 2.7 |
|  | 5 | Condition, Verbal memory, Visual memory, Visual motor speed, Reaction time, Condition x Verbal memory, Condition x Visual memory, Condition x Visual motor speed, Condition x Reaction time | Intercepts & Slopes | 587.8 | 626.4 | -279.9 | 3 | 14 | | 1.0 |
| Pelvis rotation [°] | 1 | - | - | 847.7 | 853.2 | -421.9 | - | - | | - |
|  | 2 | - | Intercepts | 769.9 | 778.2 | -381.9 | 1 | 3 | | 79.8** |
|  | 3 | Condition, Verbal memory, Visual memory, Visual motor speed, Reaction time, Condition x Verbal memory, Condition x Visual memory, Condition x Visual motor speed, Condition x Reaction time | Intercepts | 767.9 | 801.0 | -372.0 | 2 | 12 | | 20.0* |
|  | 4 | Condition, Verbal memory, Visual memory, Visual motor speed, Reaction time, Condition x Verbal memory, Condition x Visual memory, Condition x Visual motor speed, Condition x Reaction time, Expertise | Intercepts | 769.8 | 805.6 | -371.9 | 3 | 13 | | 0.2 |
|  | 5 | Condition, Verbal memory, Visual memory, Visual motor speed, Reaction time, Condition x Verbal memory, Condition x Visual memory, Condition x Visual motor speed, Condition x Reaction time | Intercepts & Slopes | 770.9 | 809.4 | -371.4 | 3 | 14 | | 1.0 |
| Hip abduction [°] | 1 | - | - | 696.9 | 702.4 | -346.4 | - | - | | - |
|  | 2 | - | Intercepts | 585.6 | 593.8 | -289.8 | 1 | 3 | | 113.3** |
|  | 3 | Condition, Verbal memory, Visual memory, Visual motor speed, Reaction time, Condition x Verbal memory, Condition x Visual memory, Condition x Visual motor speed, Condition x Reaction time | Intercepts | 599.2 | 632.2 | -287.6 | 2 | 12 | | 4.4 |
|  | 4 | Condition, Verbal memory, Visual memory, Visual motor speed, Reaction time, Condition x Verbal memory, Condition x Visual memory, Condition x Visual motor speed, Condition x Reaction time, Expertise | Intercepts | 600.8 | 636.6 | -287.4 | 3 | 13 | | 0.4 |
|  | 5 | Condition, Verbal memory, Visual memory, Visual motor speed, Reaction time, Condition x Verbal memory, Condition x Visual memory, Condition x Visual motor speed, Condition x Reaction time | Intercepts & Slopes | 603.2 | 641.7 | -287.6 | 3 | 14 | | 0.01 |
| Hip rotation [°] | 1 | - | - | 871.5 | 877.0 | -433.8 | - | - | | - |
|  | 2 | - | Intercepts | 734.7 | 742.9 | -364.3 | 1 | 3 | | 138.8** |
|  | 3 | Condition, Verbal memory, Visual memory, Visual motor speed, Reaction time, Condition x Verbal memory, Condition x Visual memory, Condition x Visual motor speed, Condition x Reaction time | Intercepts | 727.7 | 760.7 | -351.8 | 2 | 12 | | 25.0* |
|  | 4 | Condition, Verbal memory, Visual memory, Visual motor speed, Reaction time, Condition x Verbal memory, Condition x Visual memory, Condition x Visual motor speed, Condition x Reaction time, Expertise | Intercepts | 728.6 | 764.4 | -351.3 | 3 | 13 | | 1.1 |
|  | 5 | Condition, Verbal memory, Visual memory, Visual motor speed, Reaction time, Condition x Verbal memory, Condition x Visual memory, Condition x Visual motor speed, Condition x Reaction time | Intercepts & Slopes | 730.7 | 769.2 | -351.3 | 3 | 14 | | 1.0 |
| Knee flexion [°] | 1 | - | - | 793.6 | 799.1 | -394.8 | - | - | | - |
|  | 2 | - | Intercepts | 711.7 | 719.9 | -352.8 | 1 | 3 | | 84.0** |
|  | 3 | Condition, Verbal memory, Visual memory, Visual motor speed, Reaction time, Condition x Verbal memory, Condition x Visual memory, Condition x Visual motor speed, Condition x Reaction time | Intercepts | 719.6 | 752.6 | -347.8 | 2 | 12 | | 10.1 |
|  | 4 | Condition, Verbal memory, Visual memory, Visual motor speed, Reaction time, Condition x Verbal memory, Condition x Visual memory, Condition x Visual motor speed, Condition x Reaction time, Expertise | Intercepts | 720.3 | 756.1 | -347.1 | 3 | 13 | | 1.3 |
|  | 5 | Condition, Verbal memory, Visual memory, Visual motor speed, Reaction time, Condition x Verbal memory, Condition x Visual memory, Condition x Visual motor speed, Condition x Reaction time | Intercepts & Slopes | 723.6 | 762.1 | -347.8 | 3 | 14 | | 0.005 |
| Knee abduction [°] | 1 | - | - | 634.4 | 639.9 | -315.2 | - | - | | - |
|  | 2 | - | Intercepts | 452.3 | 460.6 | -223.1 | 1 | 3 | | 184.1** |
|  | 3 | Condition, Verbal memory, Visual memory, Visual motor speed, Reaction time, Condition x Verbal memory, Condition x Visual memory, Condition x Visual motor speed, Condition x Reaction time | Intercepts | 461.2 | 494.3 | -218.6 | 2 | 12 | | 9.1 |
|  | 4 | Condition, Verbal memory, Visual memory, Visual motor speed, Reaction time, Condition x Verbal memory, Condition x Visual memory, Condition x Visual motor speed, Condition x Reaction time, Expertise | Intercepts | 463.0 | 498.8 | -218.5 | 3 | 13 | | 0.3 |
|  | 5 | Condition, Verbal memory, Visual memory, Visual motor speed, Reaction time, Condition x Verbal memory, Condition x Visual memory, Condition x Visual motor speed, Condition x Reaction time | Intercepts & Slopes | 462.9 | 501.5 | -217.5 | 3 | 14 | | 2.3 |
| Step width (cm) | 1 | - | - | 151.2 | 156.7 | -73.6 | - | - | | - |
|  | 2 | - | Intercepts | 83.9 | 92.2 | -39.0 | 1 | 3 | | 69.3** |
|  | 3 | Condition, Verbal memory, Visual memory, Visual motor speed, Reaction time, Condition x Verbal memory, Condition x Visual memory, Condition x Visual motor speed, Condition x Reaction time | Intercepts | 78.5 | 111.5 | -27.2 | 2 | 12 | | 23.4* |
|  | 4 | Condition, Verbal memory, Visual memory, Visual motor speed, Reaction time, Condition x Verbal memory, Condition x Visual memory, Condition x Visual motor speed, Condition x Reaction time, Expertise | Intercepts | 80.4 | 116.2 | -27.2 | 3 | 13 | | 0.07 |
|  | 5 | Condition, Verbal memory, Visual memory, Visual motor speed, Reaction time, Condition x Verbal memory, Condition x Visual memory, Condition x Visual motor speed, Condition x Reaction time | Intercepts & Slopes | 82.2 | 120.8 | -27.1 | 3 | 14 | | 0.3 |
| * p < 0.05, ** p < 0.001 | | | | | | | | | | |

**Supplement C: Results of Correlation Analyses among ImPACT Composite Scores**

|  | Verbal memory | Visual memory | Visual motor speed | Reaction time |
| --- | --- | --- | --- | --- |
| Verbal memory | - |  |  |  |
| Visual memory | 0.27 | - |  |  |
| Visual motor speed | 0.20 | 0.67* | - |  |
| Reaction time | 0.06 | -0.13 | -0.15 | - |
| Values represent Pearson correlation coefficients (r); * p < 0.001 | | | | |

**Supplement D: Linear mixed models outputs for kinematics at penultimate step foot strike (pen-FS).**

| **Dependent variable** | **Predictors** | **Beta (β)** | **SE β** | **95% CI** | **t** | **p** | **marg. R^2^** | **cond. R^2^** | **ICC** |
| --- | --- | --- | --- | --- | --- | --- | --- | --- | --- |
| Lateral trunk flexion [°] | Intercept | -3.49 | 0.66 | -4.74, -2.23 | -5.30 | <0.001 | 0.31 | 0.78 | 0.69 |
|  | Condition | **1.00** | **0.17** | **0.67, 1.33** | **5.78** | **<0.001** |  |  |  |
|  | Verbal memory | 1.19 | 0.70 | -0.19, 2.57 | 1.70 | 0.10 |  |  |  |
|  | Visual memory | -0.05 | 0.92 | -1.86, 1.76 | -0.05 | 0.96 |  |  |  |
|  | Visual motor speed | **-2.20** | **0.90** | **-3.97, -0.42** | **-2.44** | **0.02** |  |  |  |
|  | Reaction time | 1.27 | 0.68 | -0.08, 2.61 | 1.86 | 0.08 |  |  |  |
|  | Condition x Verbal memory | **-0.52** | **0.18** | **-0.88, -0.17** | **-2.84** | **0.006** |  |  |  |
|  | Condition x Visual memory | -0.09 | 0.24 | -0.56, 0.37 | -0.39 | 0.70 |  |  |  |
|  | Condition x Visual motor speed | 0.24 | 0.24 | -0.22, 0.69 | 1.00 | 0.32 |  |  |  |
|  | Condition x Reaction time | **-0.36** | **0.18** | **-0.70, -0.02** | **-2.01** | **0.047** |  |  |  |
|  | Expertise | -0.85 | 1.33 | -3.46, 1.76 | -0.64 | 0.53 |  |  |  |
| Trunk rotation [°] | Intercept | 12.45 | 1.50 | 9.59, 15.30 | 8.29 | <0.001 | 0.09 | 0.71 | 0.68 |
|  | Condition | **-0.69** | **0.33** | **-1.31, -0.08** | **-2.13** | **0.04** |  |  |  |
|  | Verbal memory | -1.38 | 1.60 | -4.54, 1.77 | -0.87 | 0.40 |  |  |  |
|  | Visual memory | -0.91 | 2.09 | -5.04, 3.22 | -0.43 | 0.67 |  |  |  |
|  | Visual motor speed | 1.30 | 2.06 | -2.76, 5.36 | 0.63 | 0.53 |  |  |  |
|  | Reaction time | -0.26 | 1.56 | -3.33, 2.81 | -0.17 | 0.87 |  |  |  |
|  | Condition x Verbal memory | 0.17 | 0.35 | -0.49, 0.83 | 0.50 | 0.62 |  |  |  |
|  | Condition x Visual memory | -0.15 | 0.45 | -1.01, 0.71 | -0.33 | 0.74 |  |  |  |
|  | Condition x Visual motor speed | 0.31 | 0.45 | -0.53, 1.16 | 0.70 | 0.48 |  |  |  |
|  | Condition x Reaction time | 0.09 | 0.34 | -0.55, 0.73 | 0.27 | 0.79 |  |  |  |
|  | Expertise | -4.15 | 2.10 | -8.29, -0.02 | -1.98 | 0.06 |  |  |  |
| Frontal pelvis tilt [°] | Intercept | -10.50 | 0.71 | -11.84, -9.16 | -14.88 | <0.001 | 0.30 | 0.85 | 0.79 |
|  | Condition | **0.78** | **0.16** | **0.47, 1.10** | **4.79** | **<0.001** |  |  |  |
|  | Verbal memory | **1.85** | **0.75** | **0.37, 3.33** | 2.46 | **0.02** |  |  |  |
|  | Visual memory | 0.04 | 0.98 | -1.90, 1.98 | 0.04 | 0.97 |  |  |  |
|  | Visual motor speed | -0.66 | 0.97 | -2.56, 1.25 | -0.68 | 0.50 |  |  |  |
|  | Reaction time | **1.99** | **0.73** | **0.55, 3.44** | **2.73** | **0.01** |  |  |  |
|  | Condition x Verbal memory | **-0.44** | **0.17** | **-0.78, -0.11** | **-2.55** | **0.01** |  |  |  |
|  | Condition x Visual memory | 0.37 | 0.23 | -0.06, 0.81 | 1.63 | 0.11 |  |  |  |
|  | Condition x Visual motor speed | -0.24 | 0.22 | -0.66, 0.19 | -1.05 | 0.30 |  |  |  |
|  | Condition x Reaction time | -0.03 | 0.17 | -0.35, 0.29 | -0.17 | 0.87 |  |  |  |
|  | Expertise | -2.96 | 1.46 | -5.83, -0.09 | -2.03 | 0.05 |  |  |  |
| Pelvis rotation [°] | Intercept | 12.62 | 1.59 | 9.60, 15.65 | 7.93 | <0.001 | 0.21 | 0.67 | 0.59 |
|  | Condition | -0.36 | 0.53 | -1.37, 0.65 | -0.68 | 0.50 |  |  |  |
|  | Verbal memory | -3.07 | 1.69 | -6.41, 0.27 | -1.81 | 0.08 |  |  |  |
|  | Visual memory | -0.89 | 2.22 | -5.27, 3.49 | -0.40 | 0.69 |  |  |  |
|  | Visual motor speed | 2.85 | 2.18 | -1.45, 7.16 | 1.31 | 0.20 |  |  |  |
|  | Reaction time | -0.20 | 1.65 | -3.46, 3.05 | -0.12 | 0.90 |  |  |  |
|  | Condition x Verbal memory | 0.30 | 0.56 | -0.77, 1.37 | 0.53 | 0.60 |  |  |  |
|  | Condition x Visual memory | -0.61 | 0.74 | -2.01, 0.80 | -0.82 | 0.41 |  |  |  |
|  | Condition x Visual motor speed | 0.33 | 0.73 | -1.05, 1.71 | 0.45 | 0.65 |  |  |  |
|  | Condition x Reaction time | -0.16 | 0.55 | -1.21, 0.88 | -0.30 | 0.77 |  |  |  |
|  | Expertise | -1.20 | 2.53 | -6.18, 3.78 | -0.47 | 0.64 |  |  |  |
| Step width [cm] | Intercept | 25.11 | 0.84 | 23.51, 26.71 | 29.81 | <0.001 | 0.13 | 0.60 | 0.54 |
|  | Condition | **-0.80** | **0.29** | **-1.35, -0.26** | **-2.79** | **0.01** |  |  |  |
|  | Verbal memory | -0.19 | 0.90 | -1.96, 1.58 | -0.21 | 0.84 |  |  |  |
|  | Visual memory | 0.41 | 1.17 | -1.90, 2.73 | 0.35 | 0.73 |  |  |  |
|  | Visual motor speed | -0.37 | 1.15 | -2.65, 1.91 | -0.32 | 0.75 |  |  |  |
|  | Reaction time | 0.20 | 0.87 | -1.52, 1.92 | 0.23 | 0.82 |  |  |  |
|  | Condition x Verbal memory | -0.05 | 0.31 | -0.63, 0.54 | -0.15 | 0.88 |  |  |  |
|  | Condition x Visual memory | -0.75 | 0.40 | -1.51, 0.01 | -1.87 | 0.07 |  |  |  |
|  | Condition x Visual motor speed | 0.50 | 0.39 | -0.25, 1.25 | 1.26 | 0.21 |  |  |  |
|  | Condition x Reaction time | -0.32 | 0.30 | -0.88, 0.25 | -1.07 | 0.29 |  |  |  |
|  | Expertise | 1.34 | 1.55 | -1.71, 4.39 | 0.86 | 0.40 |  |  |  |

**Supplement E: Linear mixed models outputs for kinematics at ultimate step foot strike (ult-FS).**

| **Dependent variable** | **Predictors** | **Beta (β)** | **SE β** | **95% CI** | **t** | **p** | **marg. R^2^** | **cond. R^2^** | **ICC** |
| --- | --- | --- | --- | --- | --- | --- | --- | --- | --- |
| Lateral trunk flexion [°] | Intercept | -2.96 | 0.68 | -4.26, -1.66 | -4.34 | <0.001 | 0.26 | 0.79 | 0.71 |
|  | Condition | **0.76** | **0.18** | **0.42, 1.11** | **4.19** | **<0.001** |  |  |  |
|  | Verbal memory | 1.41 | 0.73 | -0.02, 2.84 | 1.94 | 0.06 |  |  |  |
|  | Visual memory | -0.26 | 0.95 | -2.14, 1.61 | -0.28 | 0.78 |  |  |  |
|  | Visual motor speed | -0.63 | 0.93 | -2.48, 1.21 | -0.68 | 0.50 |  |  |  |
|  | Reaction time | **2.18** | **0.71** | **0.78, 3.57** | **3.08** | **0.005** |  |  |  |
|  | Condition x Verbal memory | -0.35 | 0.19 | -0.72, 0.02 | -1.78 | 0.08 |  |  |  |
|  | Condition x Visual memory | 0.08 | 0.25 | -0.41, 0.56 | 0.30 | 0.77 |  |  |  |
|  | Condition x Visual motor speed | -0.03 | 0.25 | -0.51, 0.44 | -0.13 | 0.90 |  |  |  |
|  | Condition x Reaction time | -0.19 | 0.19 | -0.55, 0.17 | -0.98 | 0.33 |  |  |  |
|  | Expertise | -1.79 | 1.40 | -4.55, 0.96 | -1.28 | 0.21 |  |  |  |
| Trunk rotation [°] | Intercept | 3.78 | 2.21 | -0.41, 7.98 | 1.71 | 0.090 | 0.08 | 0.78 | 0.76 |
|  | Condition | -0.19 | 0.43 | -1.00, 0.62 | -0.45 | 0.66 |  |  |  |
|  | Verbal memory | -2.10 | 2.35 | -6.73, 2.53 | -0.90 | 0.38 |  |  |  |
|  | Visual memory | -0.20 | 3.08 | -6.28, 5.87 | -0.07 | 0.95 |  |  |  |
|  | Visual motor speed | -1.23 | 3.02 | -7.20, 4.73 | -0.41 | 0.69 |  |  |  |
|  | Reaction time | -2.22 | 2.29 | -6.73, 2.29 | -0.97 | 0.34 |  |  |  |
|  | Condition x Verbal memory | 0.54 | 0.45 | -0.32, 1.40 | 1.19 | 0.24 |  |  |  |
|  | Condition x Visual memory | -0.12 | 0.60 | -1.25, 1.01 | -0.20 | 0.84 |  |  |  |
|  | Condition x Visual motor speed | 0.91 | 0.58 | -0.20, 2.02 | 1.55 | 0.13 |  |  |  |
|  | Condition x Reaction time | 0.15 | 0.44 | -0.69, 0.99 | 0.35 | 0.73 |  |  |  |
|  | Expertise | -4.50 | 2.94 | -10.29, 1.29 | -1.53 | 0.14 |  |  |  |
| Frontal pelvis tilt [°] | Intercept | -11.45 | 0.81 | -12.99, -9.91 | -14.13 | <0.001 | 0.29 | 0.87 | 0.81 |
|  | Condition | **0.55** | **0.17** | **0.23, 0.87** | **3.29** | **0.001** |  |  |  |
|  | Verbal memory | 1.25 | 0.86 | -0.45, 2.96 | 1.45 | 0.16 |  |  |  |
|  | Visual memory | 1.66 | 1.13 | -0.57, 3.89 | 1.47 | 0.15 |  |  |  |
|  | Visual motor speed | -1.96 | 1.11 | -4.16, 0.23 | -1.77 | 0.09 |  |  |  |
|  | Reaction time | **2.29** | **0.84** | **0.64, 3.95** | **2.73** | **0.01** |  |  |  |
|  | Condition x Verbal memory | -0.15 | 0.18 | -0.49, 0.19 | -0.86 | 0.39 |  |  |  |
|  | Condition x Visual memory | -0.01 | 0.23 | -0.45, 0.44 | -0.03 | 0.98 |  |  |  |
|  | Condition x Visual motor speed | 0.00 | 0.23 | -0.43, 0.44 | 0.02 | 0.99 |  |  |  |
|  | Condition x Reaction time | -0.12 | 0.17 | -0.45, 0.21 | -0.70 | 0.48 |  |  |  |
|  | Expertise | -2.48 | 1.71 | -5.86, 0.89 | -1.45 | 0.16 |  |  |  |
| Pelvis rotation [°] | Intercept | 23.64 | 1.92 | 19.99, 27.29 | 12.31 | <0.001 | 0.14 | 0.78 | 0.75 |
|  | Condition | **-1.17** | **0.39** | **-1.91, -0.44** | **-3.02** | **0.003** |  |  |  |
|  | Verbal memory | -3.13 | 2.04 | -7.16, 0.90 | -1.53 | 0.14 |  |  |  |
|  | Visual memory | -1.42 | 2.68 | -6.71, 3.86 | -0.53 | 0.60 |  |  |  |
|  | Visual motor speed | 0.84 | 2.63 | -4.35, 6.04 | 0.32 | 0.75 |  |  |  |
|  | Reaction time | -2.64 | 1.99 | -6.57, 1.29 | -1.33 | 0.20 |  |  |  |
|  | Condition x Verbal memory | 0.70 | 0.41 | -0.09, 1.48 | 1.69 | 0.10 |  |  |  |
|  | Condition x Visual memory | 0.29 | 0.54 | -0.74, 1.32 | 0.54 | 0.59 |  |  |  |
|  | Condition x Visual motor speed | 0.28 | 0.53 | -0.73, 1.29 | 0.52 | 0.60 |  |  |  |
|  | Condition x Reaction time | 0.18 | 0.40 | -0.59, 0.94 | 0.44 | 0.66 |  |  |  |
|  | Expertise | 2.36 | 3.37 | -4.27, 8.99 | 0.70 | 0.49 |  |  |  |
| Hip abduction [°] | Intercept | 9.00 | 0.95 | 7.19, 10.80 | 9.48 | <0.001 | 0.05 | 0.83 | 0.82 |
|  | Condition | 0.24 | 0.17 | -0.09, 0.57 | 1.38 | 0.17 |  |  |  |
|  | Verbal memory | 0.94 | 1.01 | -1.05, 2.94 | 0.93 | 0.36 |  |  |  |
|  | Visual memory | -0.35 | 1.32 | -2.96, 2.26 | -0.26 | 0.80 |  |  |  |
|  | Visual motor speed | 0.33 | 1.30 | -2.23, 2.90 | 0.26 | 0.80 |  |  |  |
|  | Reaction time | 0.06 | 0.98 | -1.88, 2.00 | 0.06 | 0.95 |  |  |  |
|  | Condition x Verbal memory | -0.02 | 0.18 | -0.37, 0.33 | -0.10 | 0.92 |  |  |  |
|  | Condition x Visual memory | 0.09 | 0.24 | -0.37, 0.55 | 0.38 | 0.71 |  |  |  |
|  | Condition x Visual motor speed | 0.02 | 0.24 | -0.43, 0.48 | 0.10 | 0.92 |  |  |  |
|  | Condition x Reaction time | -0.10 | 0.18 | -0.44, 0.24 | -0.56 | 0.58 |  |  |  |
|  | Expertise | -1.13 | 1.95 | -4.97, 2.71 | -0.58 | 0.57 |  |  |  |
| Hip internal rotation [°] | Intercept | 4.16 | 1.63 | 1.07, 7.26 | 2.56 | 0.012 | 0.29 | 0.89 | 0.85 |
|  | Condition | **0.87** | **0.31** | **0.29, 1.46** | **2.83** | **0.01** |  |  |  |
|  | Verbal memory | 2.93 | 1.73 | -0.49, 6.35 | 1.69 | 0.10 |  |  |  |
|  | Visual memory | 2.86 | 2.27 | -1.62, 7.34 | 1.26 | 0.22 |  |  |  |
|  | Visual motor speed | -0.90 | 2.23 | -5.30, 3.50 | -0.41 | 0.69 |  |  |  |
|  | Reaction time | **3.90** | **1.69** | **0.57, 7.23** | **2.31** | **0.03** |  |  |  |
|  | Condition x Verbal memory | -0.25 | 0.33 | -0.87, 0.37 | -0.77 | 0.45 |  |  |  |
|  | Condition x Visual memory | -0.53 | 0.43 | -1.35, 0.29 | -1.24 | 0.22 |  |  |  |
|  | Condition x Visual motor speed | -0.02 | 0.42 | -0.83, 0.78 | -0.06 | 0.95 |  |  |  |
|  | Condition x Reaction time | 0.25 | 0.32 | -0.35, 0.86 | 0.80 | 0.43 |  |  |  |
|  | Expertise | -5.31 | 3.46 | -12.13, 1.50 | -1.54 | 0.14 |  |  |  |
| Knee flexion [°] | Intercept | 27.23 | 1.40 | 24.56, 29.90 | 19.41 | <0.001 | 0.14 | 0.76 | 0.72 |
|  | Condition | 0.18 | 0.31 | -0.41, 0.77 | 0.58 | 0.56 |  |  |  |
|  | Verbal memory | -1.69 | 1.49 | -4.63, 1.26 | -1.13 | 0.27 |  |  |  |
|  | Visual memory | -1.13 | 1.96 | -4.99, 2.73 | -0.58 | 0.57 |  |  |  |
|  | Visual motor speed | 0.36 | 1.92 | -3.43, 4.16 | 0.19 | 0.85 |  |  |  |
|  | Reaction time | -0.58 | 1.45 | -3.45, 2.29 | -0.40 | 0.69 |  |  |  |
|  | Condition x Verbal memory | 0.05 | 0.33 | -0.58, 0.68 | 0.16 | 0.87 |  |  |  |
|  | Condition x Visual memory | -0.28 | 0.43 | -1.10, 0.55 | -0.64 | 0.53 |  |  |  |
|  | Condition x Visual motor speed | 0.79 | 0.43 | -0.02, 1.60 | 1.85 | 0.07 |  |  |  |
|  | Condition x Reaction time | -0.13 | 0.32 | -0.74, 0.48 | -0.40 | 0.69 |  |  |  |
|  | Expertise | 2.94 | 2.65 | -2.28, 8.16 | 1.11 | 0.28 |  |  |  |
| Knee abduction [°] | Intercept | 7.53 | 0.67 | 6.26, 8.79 | 11.29 | <0.001 | 0.16 | 0.93 | 0.91 |
|  | Condition | 0.15 | 0.09 | -0.02, 0.31 | 1.70 | 0.09 |  |  |  |
|  | Verbal memory | -0.76 | 0.71 | -2.16, 0.64 | -1.08 | 0.29 |  |  |  |
|  | Visual memory | 1.44 | 0.93 | -0.39, 3.28 | 1.55 | 0.13 |  |  |  |
|  | Visual motor speed | -1.70 | 0.91 | -3.50, 0.10 | -1.86 | 0.08 |  |  |  |
|  | Reaction time | -0.18 | 0.69 | -1.54, 1.18 | -0.26 | 0.79 |  |  |  |
|  | Condition x Verbal memory | -0.02 | 0.09 | -0.19, 0.16 | -0.19 | 0.85 |  |  |  |
|  | Condition x Visual memory | -0.04 | 0.12 | -0.27, 0.19 | -0.34 | 0.74 |  |  |  |
|  | Condition x Visual motor speed | 0.01 | 0.12 | -0.21, 0.24 | 0.11 | 0.92 |  |  |  |
|  | Condition x Reaction time | 0.05 | 0.09 | -0.12, 0.22 | 0.59 | 0.56 |  |  |  |
|  | Expertise | 0.71 | 1.46 | -2.16, 3.58 | 0.49 | 0.63 |  |  |  |
| Step width [cm] | Intercept | 26.36 | 0.91 | 24.64, 28.08 | 29.10 | <0.001 | 0.08 | 0.77 | 0.75 |
|  | Condition | **0.79** | **0.19** | **0.42, 1.15** | **4.05** | **<0.001** |  |  |  |
|  | Verbal memory | 1.24 | 0.96 | -0.66, 3.14 | 1.28 | 0.21 |  |  |  |
|  | Visual memory | 0.99 | 1.26 | -1.50, 3.48 | 0.78 | 0.44 |  |  |  |
|  | Visual motor speed | -0.12 | 1.24 | -2.57, 3.32 | -0.10 | 0.92 |  |  |  |
|  | Reaction time | 0.73 | 0.94 | -1.12, 2.58 | 0.78 | 0.44 |  |  |  |
|  | Condition x Verbal memory | -0.28 | 0.21 | -0.67, 0.12 | -1.34 | 0.19 |  |  |  |
|  | Condition x Visual memory | -0.40 | 0.27 | -0.91, 0.12 | -1.47 | 0.15 |  |  |  |
|  | Condition x Visual motor speed | 0.04 | 0.27 | -0.46, 0.55 | 0.16 | 0.87 |  |  |  |
|  | Condition x Reaction time | -0.13 | 0.20 | -0.51, 0.25 | -0.64 | 0.52 |  |  |  |
|  | Expertise | 0.45 | 1.78 | -3.04, 3.95 | 0.25 | 0.80 |  |  |  |
